# A comprehensive illustrated protocol for clearing, mounting, and imaging leaf venation networks

**DOI:** 10.1002/aps3.70002

**Published:** 2025-03-07

**Authors:** Isabella Niewiadomski, Monica Antonio, Luiza Maria T. Aparecido, Mickey Boakye, Sonoma Carlos, Andrea Echevarria, Adrian Fontao, Joseph Mann, Ilaíne Silveira Matos, Norma Salinas, Bradley Vu, Benjamin Wong Blonder

**Affiliations:** ^1^ Department of Environmental Science, Policy, and Management University of California at Berkeley Berkeley California USA; ^2^ School of Biological Sciences University of Utah Salt Lake City Utah USA; ^3^ School of Biological Sciences The University of Adelaide Adelaide South Australia Australia; ^4^ Department of Science, Chemistry Section, and Institute for Nature Earth and Energy Pontifical Catholic University of Peru Lima Peru

**Keywords:** cleared leaf, histology, leaf architecture, leaf venation network, vascular network

## Abstract

**Premise:**

Leaf venation network architecture can provide insights into plant evolution, ecology, and physiology. Venation networks are typically assessed through histological methods, but existing protocols provide limited guidance on processing large or challenging leaves.

**Methods and results:**

We present an illustrated protocol for visualizing whole leaf venation networks, including sample preparation, clearing, staining, mounting, imaging, and archiving steps. The protocol also includes supply lists, troubleshooting procedures, safety considerations, and examples of successful and unsuccessful outcomes. The protocol is suitable for a wide range of leaf sizes and morphologies and has been used with all major plant groups.

**Conclusion:**

We provide a workflow for obtaining high‐quality mounts and images of venation networks of a wide range of species, using readily available materials.

Venation networks provide many functions in leaves, including mechanical support, damage resistance, water transport, and sugar transport (Roth‐Nebelsick et al., 2001; Sack and Scoffoni, 2013). These networks show a large diversity of variation in form, ranging from branching to reticulate patterns, and from single levels of patterning to multiple hierarchical levels (Ellis et al., 2009). This diversity of form and function has made venation networks relevant to a range of questions in plant evolution (Roth‐Nebelsick et al., 2001; Boyce et al., 2009), ecophysiology (Matos et al., 2024), development (Candela et al., 1999), and ecology (Sack and Scoffoni, 2013; Blonder et al., 2017), as well as in paleoclimate reconstruction (Uhl and Mosbrugger, 1999; Blonder et al., 2017; Peppe et al., 2018). Thus, reliable methods are needed to produce high‐quality images of leaf venation networks across a wide variety of plant species.

There is a long history of method development for clearing and mounting leaves to visualize their leaf venation networks (Dilcher, 1974). Early methods relied on water digestion (Seba, 1730) and mechanical scrubbing (Parrish, 1862), or on the application of pressure to make contact prints (von Ettingshausen, 1861). Leaf tissue can be cleared using acids (Ram and Nayyar, 1978; García‐Gutiérrez et al., 2020) or bases (Peace, 1910; Morley, 1949; Fuchs, 1963; Dizeo de Strittmatter, 1973; Pérez‐Harguindeguy et al., 2013), sometimes with the addition of chloral hydrate (Peace, 1910; Candela et al., 1999; Lux et al., 2015) and/or phenol (Keane et al., 1988). Contrast is achieved using a range of stains including safranin (Peace, 1910; Pérez‐Harguindeguy et al., 2013; Vasco et al., 2014), toluidine blue (Kuo et al., 1972; Brodribb et al., 2007; García‐Gutiérrez et al., 2020), acid fuchsin (Fuchs, 1963), and fast green (Schadel and Dickison, 1979; Sack et al., 2012; Scoffoni and Sack, 2013). Specialized mounting protocols have also been developed, relying on a range of chemistries (Christophel and Blackburn, 1975; Buechler, 2004; Vasco et al., 2014). Non‐destructive X‐ray imaging methods, which do not require clearing or mounting, have also been developed (Wing, 1992; Blonder et al., 2012; Schneider et al., 2018); however, these methods remain less accessible as they require specialized equipment and training.

Several gaps remain for leaf venation network clearing, mounting, and imaging protocols. While the basic histology and chemistry are well described, the specific factors supporting an investigator's success in producing high‐quality cleared leaf samples have not been clearly described in the literature. Existing protocols have provided limited practical guidance on successfully processing challenging leaves, e.g., those that are large, tough, flimsy, or have epidermal structures like trichomes or spores. Often, investigators develop particular skills to refine their technique, but these techniques have not been described publicly. Existing protocols also provide limited visual illustration of key steps or examples of common challenges and problems, or description of associated laboratory organization considerations. Additionally, existing protocols provide limited descriptions of key safety challenges and sustainability issues, yielding the potential for substantial harm to the investigator and to the environment. Furthermore, existing protocols often assume access to costly high‐purity materials and supplies, which may not be accessible to all investigators.

Here we present an illustrated protocol for leaf preparation, chemical clearing, staining, mounting, and imaging of leaves, which addresses these gaps and complements existing protocols. The protocol has been tuned on hundreds of phylogenetically diverse species through the development of numerous datasets (Blonder and Enquist, 2014; Blonder et al., 2017, 2019, 2020; Duarte et al., 2023; Matos et al., 2024).

## METHODS AND RESULTS

### Materials

Suggested lab space is detailed in Appendix S1.1, and required materials are listed in Appendix S1.2. Some materials that can be made by hand rather than purchased are described in Appendix S1.3. The protocol requires access to a fume hood and a few meters of benchtop space. Required materials for clearing and mounting include large glass dishes with lids, histology cassettes or plastic trays, glass slides and/or acetate sheets, and personal protective equipment (PPE) for working with corrosive, flammable, and/or volatile chemicals. Imaging requires a trans‐illumination scanner, a digital camera with a macro lens, or a dissecting or brightfield microscope.

### Methods

**Figure 1:**
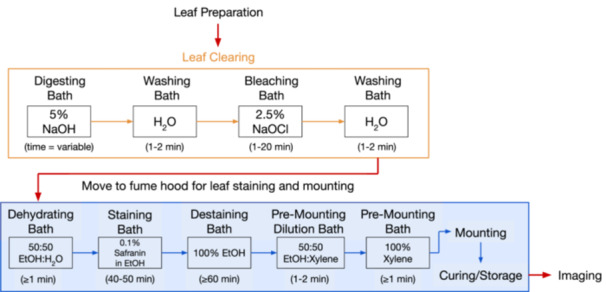
Diagram of the general workflow and timeline for sample processing in this protocol. Orange‐boxed steps occur on the benchtop; blue‐boxed steps occur in the fume hood. Solid‐line boxed text indicates a chemical bath (with adjacent text indicating processing time); unboxed text indicates a step that occurs outside of chemical baths. Red arrows indicate steps where samples are transferred to new locations.

**Figure 2:**
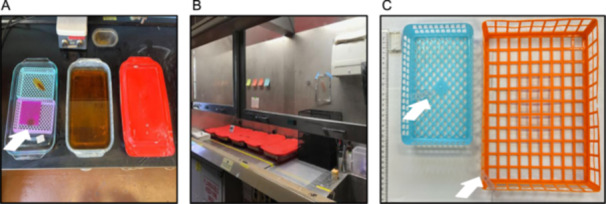
Example of typical lab setup for (A) the benchtop digestion process and (B) the fume hood staining process. (A) Example digestion baths: two with lid off, one with lid on. In the left bath, leaves are individually held in plastic cassette trays (light blue, light purple) or in a histological cassette (white). Some leaves will float when initially placed into the digestion bath, so a square piece of thin plastic cutting board (purple) is used to weigh down the leaf (indicated by arrow). In the middle bath, a large cassette is visible. The digestion bath has turned a dark amber brown, which indicates that the bath should be replaced. On the right, the chemical bath is covered with a snug lid to prevent evaporation. A hotplate used to slightly warm the baths sits above. (B) The baths are arranged in a systematic fashion from left to right according to the procedure so that handling of samples is minimized. All baths have a close‐fitting lid to prevent evaporation of volatile solvents. (C) Examples of various‐sized cassettes used to hold samples while in chemical baths. Small leaves that may fall through the holes of larger cassettes can be placed in a small white histology tissue cassette (top left next to the ruler), but most leaves can be held in a medium (middle) or large (right) plastic tray. Small plastic tags, with a species code written upon them for identification purposes, are attached to the medium and large cassettes (indicated by arrows).

The overall workflow is described in Figure 1. In brief, leaves are placed in durable cassettes, then transferred through a series of chemical baths (Figure 2). The digestion of non‐vein tissue occurs via a strong base (sodium hydroxide), with optional manipulation/agitation to slough off additional tissue. The removal of dark coloration from secondary compounds occurs via reactions with chlorine bleach (sodium hypochlorite). Staining occurs via ethanol dehydration and a safranin dye. Mounting is performed after ethanol destaining and transfer to xylene, using a xylene‐based mounting medium; mounts are prepared either between acetate sheets or glass panels.

A detailed step‐by‐step protocol is provided in Appendix S2. The protocol covers the preparation of baths, samples, and use of cassettes (Appendix S2.1); clearing via chemical digestion (Appendix S2.2); washing, bleaching, staining, and destaining (Appendix S2.3); making permanent mounts on acetate sheets or glass slides (Appendix S2.4); curing mounts and fixing mounting problems (Appendix S2.5); imaging mounts (Appendix S2.6); and providing long‐term storage of mounts (Appendix S2.7). Safety considerations are discussed in Appendix S3, covering topics such as PPE, engineering controls, and administrative controls (Appendix S3.1), as well as hazardous waste disposal (Appendix S3.2).

### Results

#### Processing rate

A typical setup might use one fume hood and two meters of bench space with eight chemical baths, with digestion accelerated via a hotplate (Figure 2). Under these conditions, samples can be processed from sample selection to mount completion in approximately one week (with the exception of very tough/thick leaves). With 20 hours of labor per week, it is reasonable to process 5–10 large whole leaves or 30–40 small leaves or leaf segments per week.

#### Key steps

Several steps of the protocol require human oversight and extra care to ensure that the timing and handling are appropriate for the condition of the sample, as small changes in timing can substantially affect the quality of outcomes. Using high‐quality samples (flat and undamaged) is also helpful. Fresh or dried leaves can both be used, with fresh leaves providing somewhat higher‐quality outcomes.

**Figure 3:**
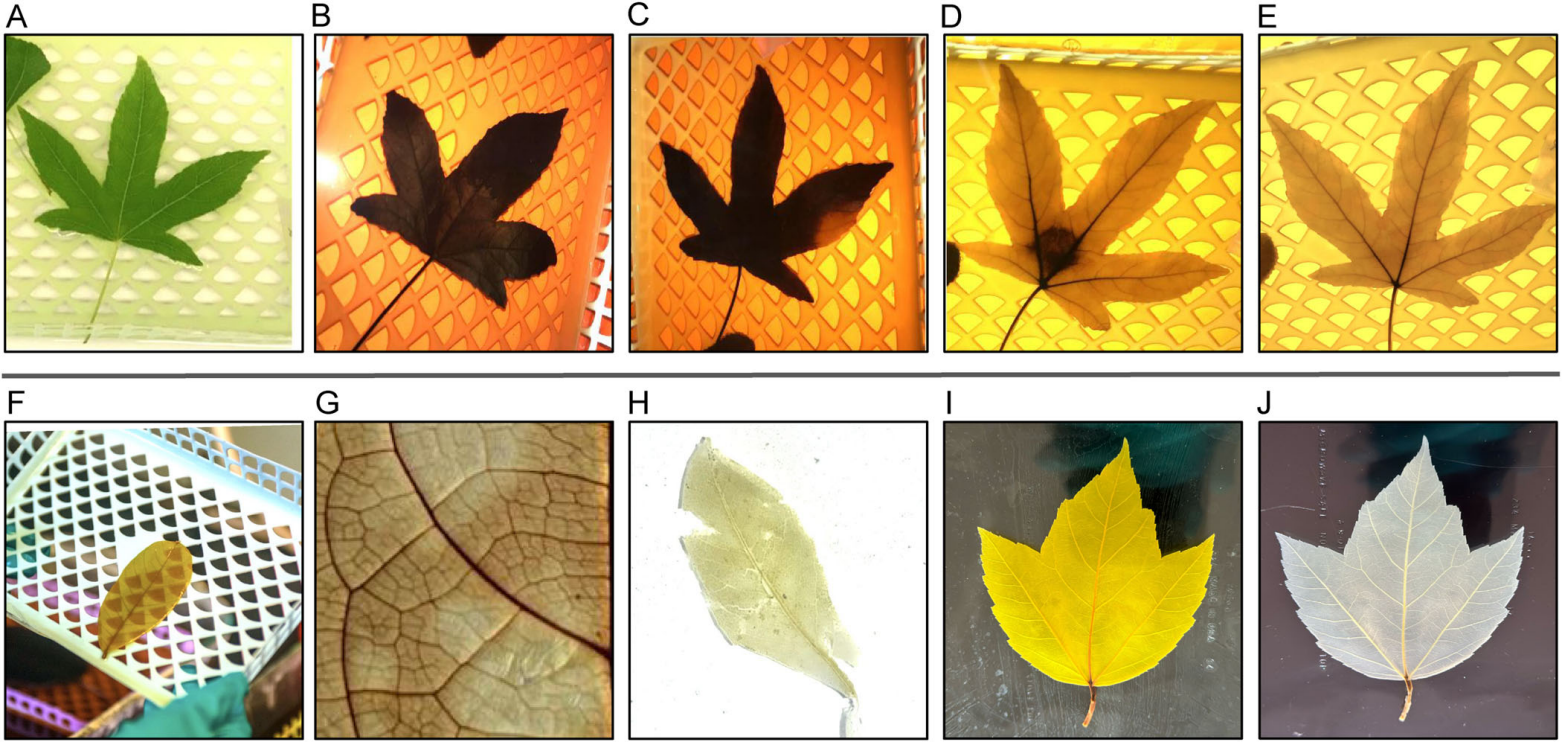
Example images illustrating leaf digestion trajectory as assessed by regular inspection. (A–E) The stages of leaf digestion for *Liquidambar styraciflua* L. (Altingiaceae). (A) A fresh leaf is held in a cassette and placed within the digestion bath. (B) The leaf sample will initially turn all black, then (C) will become slightly translucent before (D) a more transparent structure appears and (E) digestion is fully completed. The process will occur at different rates across the leaf. (F) Inspection of a leaf while it is in the digestion chemical bath, by delicately lifting it in its cassette. This avoids inadvertently tearing the leaf by lifting its petiole. (G) Example of a leaf with splotchy/uneven digestion. This leaf will need further time in the digestion bath in order to even the digestion across the entire leaf. (H) Example of a leaf that has been left in the digestion bath for too long and has begun to show signs of over‐digestion (tearing along the midrib and the leaf margins, pallid coloration). (I) *Acer rubrum* L. (Aceraceae) at the end of digestion. (J) The same leaf after being bleached.

The timing of digestion is critical. The typical progression from fresh, to dark, to semi‐transparent, to fully transparent (fully digested) can take a few days to several weeks (Figure 3A–E). The status of digestion can be checked by regularly lifting the leaf out of the digestion bath (Figure 3F). Leaves that are not ready, or that require some manual brushing or tissue sloughing, may have a blotchy appearance (Figure 3G), whereas leaves that are over‐digested and unusable have a pallid and torn appearance (Figure 3H).

Similarly, the timing of bleaching is also critical. The progression from unbleached to bleached can take a few seconds to a few hours, but typically takes ~30 seconds. A fully bleached leaf appears white, although the primary and secondary veins may retain some limited coloration (Figure 3I, J). Over‐bleaching can render a leaf unusable with a similar appearance to the leaf shown in Figure 3H.

The washing, staining, and destaining steps are more forgiving in their timing. However, transferring leaves between chemical baths must be done quickly, because the samples can be damaged by drying if substantial volatilization of solvents occurs.

**Figure 4:**
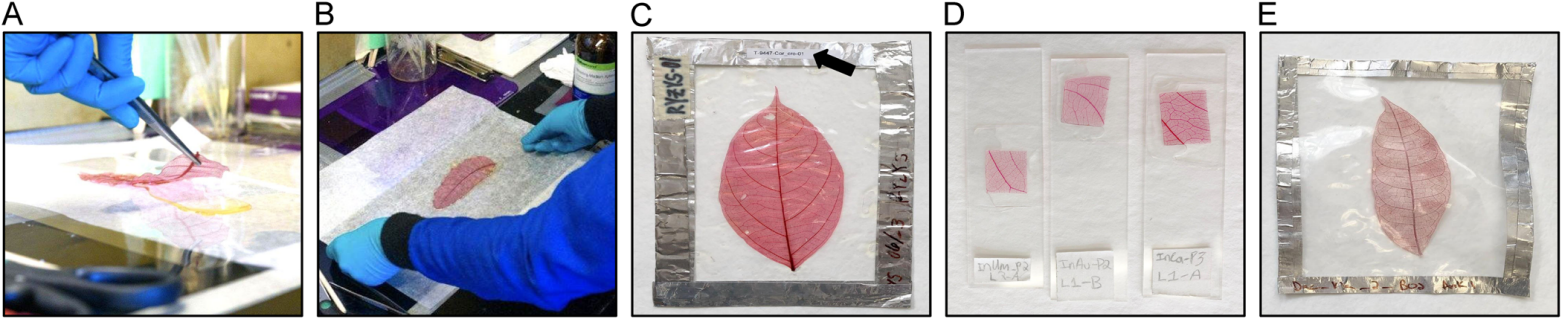
Key steps and outcomes for mounting. (A) Mounting medium has been applied to the bottom acetate sheet in a size roughly similar to the leaf to be mounted. The leaf is held at the petiole with blunt dissection tweezers and then placed upon the pool of mounting medium. Additional mounting medium is then added on top of the leaf in preparation for the top acetate sheet. (B) After the top acetate sheet is added, the two acetate sheets are pressed together in order to distribute the mounting medium evenly and tape is used to seal the edges. (C) A successful mount on acetate sheets (*Cordia croatii* J. S. Mill.; Boraginaceae). A laser‐printed label is placed on the metal sealing tape (indicated by arrow). (D) Successful mounts on glass slides (*Inga* spp.; Fabaceae). (E) A degraded mount with damage due to air bubble incursion (*Pachylobus klaineanus* Engl.; Burseraceae). This mount has low transparency and low contrast.

The mounting process requires decisive movements and attention to detail. Leaves can easily be damaged while being manipulated, solvent volatilization can still occur, and air bubbles can accidentally be introduced. The sample needs to be carefully transferred onto a glass or acetate surface with a layer of mounting medium (Figure 4A), after which additional mounting medium and another acetate or glass surface is overlaid (Figure 4B). The final mount is then flattened and optionally sealed to avoid air bubble intrusion during curing (Figure 4C, D). Insufficient mounting medium can cause air bubble intrusion, which can lead to leaf drying and low contrast (Figure 4E).

**Figure 5:**
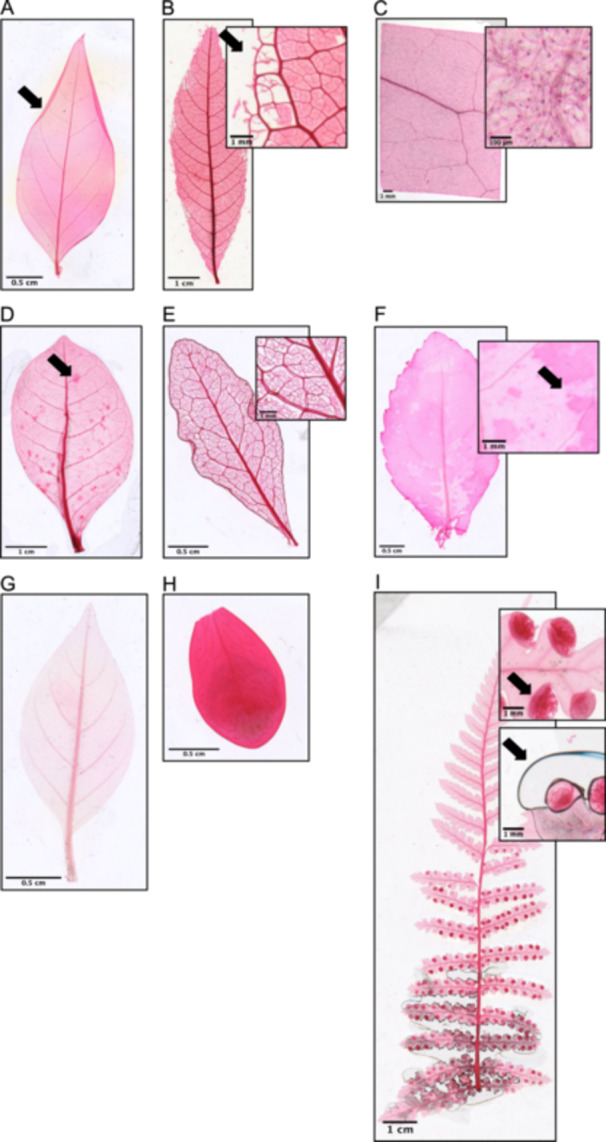
Examples of potential complications that can compromise image quality. (A) A non‐flat leaf that has folded during mounting (indicated by arrow) (*Phyllanthus purpusii* Brandegee; Phyllanthaceae). (B) Mishandling or overdigestion may cause tears (indicated by arrow) (*Ackama paniculosa* (F. Muell.) Heslewood; Cunoniaceae). (C) Trichomes that are not removed from the leaf prior to processing remain visible and obstruct visualization of venation (*Chionanthus pluriflorus* (Knobl.) Kiew; Oleaceae). (D) Pre‐existing damage (e.g., herbivory, fungal disease) remains present after clearing and will present itself as spots of uneven coloration (indicated by arrow) (*Tecomanthe speciosa* W. R. B. Oliv.; Bignoniaceae). (E) Excessive mechanical scrubbing can destroy lamina, which alters minor vein structure (indicated by white background showing through leaf) (*Euclea crispa* (Thunb.) Gürke; Ebenaceae). (F) Uneven or low contrast after staining (*Chloranthus spicatus* (Thunb.) Makino; Chloranthaceae). (G) Insufficient staining (*Cuphea nudicostata* Hemsl.; Lythraceae). (H) Excessive staining (*Lemmaphyllum microphyllum* C. Presl; Polypodiaceae). (I) Sori that are not removed will remain visible (top, indicated by arrow); air bubbles are also present (bottom, indicated by arrow) (*Dicksonia antarctica* Labill.; Cyatheaceae).

A variety of other issues can occur during the mounting process; these are listed here along with advice to avoid or repair the problem. Accidental folding (Figure 5A) is resolvable by taking more care when moving the leaf, whereas tearing (Figure 5B) can be avoided by being more delicate in handling the leaf or by shortening the digestion step. Unwanted contrast from the trichomes (Figure 5C) is resolvable by peeling/sloughing the epidermal layer during digestion. Pre‐existing damage (Figure 5D) can be addressed only by selecting healthier leaves. Micro‐tearing from scrubbing (Figure 5E) can occur but can be avoided by being gentler during the digestion step. Low contrast (Figure 5F) can sometimes be resolved by increasing the digestion time or epidermal peeling/sloughing, but in other cases cannot be resolved. Insufficient staining (Figure 5G) is resolvable by longer staining times or shorter destaining times and unresolvable otherwise, whereas excessive staining (Figure 5H) is resolvable via the opposite approach. The presence of air bubbles (Figure 5I) can be avoided by using more mounting medium or flattening the leaf prior to digestion.

**Figure 6:**
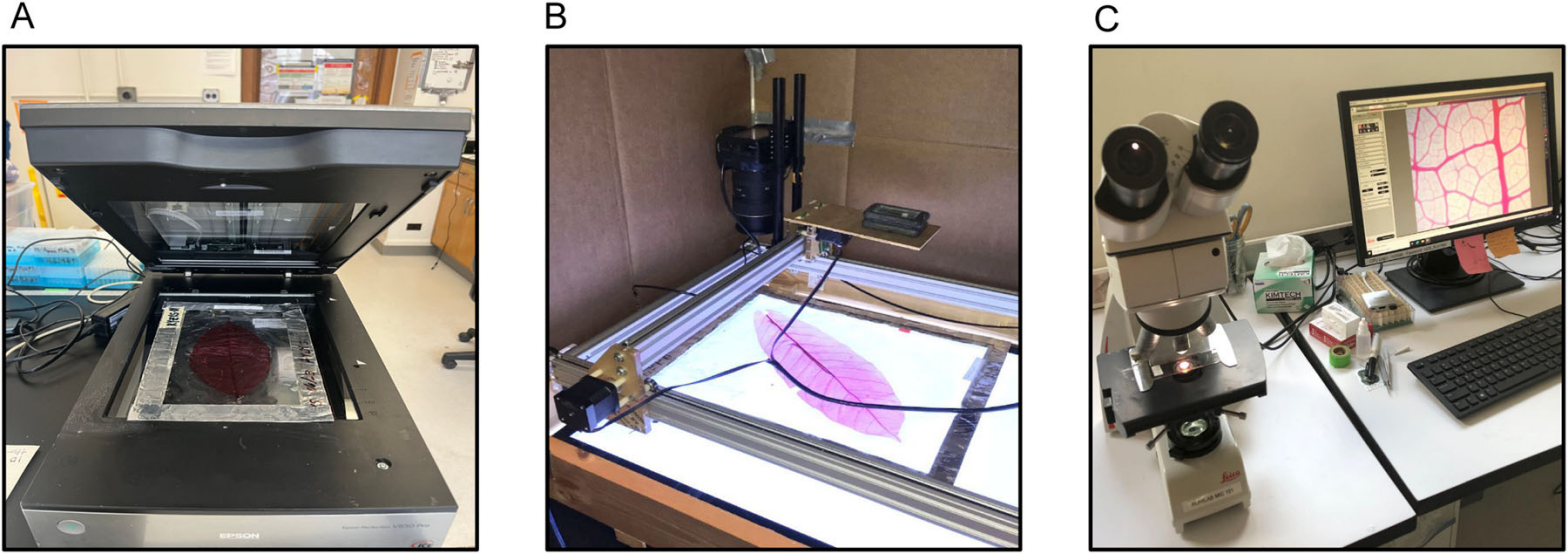
Leaf imaging methods. (A) An acetate‐mounted sample being imaged on a trans‐illumination scanner. (B) An acetate‐mounted sample being imaged by a digital camera with a macro lens attached to a computerized translation stage. (C) A slide‐mounted sample being imaged by a digital camera attached to a compound microscope.

After mount curing, the mounts can be imaged using a range of approaches. A simple option is a trans‐illuminated scanner (Figure 6A), but a digital camera with a macro lens can also work well, and a computer‐controlled translation stage and panorama software can augment this system to generate images of large leaves (Figure 6B). A brightfield or dissecting microscope with digital camera attachment (Figure 6C) can also work. It should be noted that while this option provides the highest resolution, it offers the lowest spatial extent.

#### Example results

**Figure 7:**
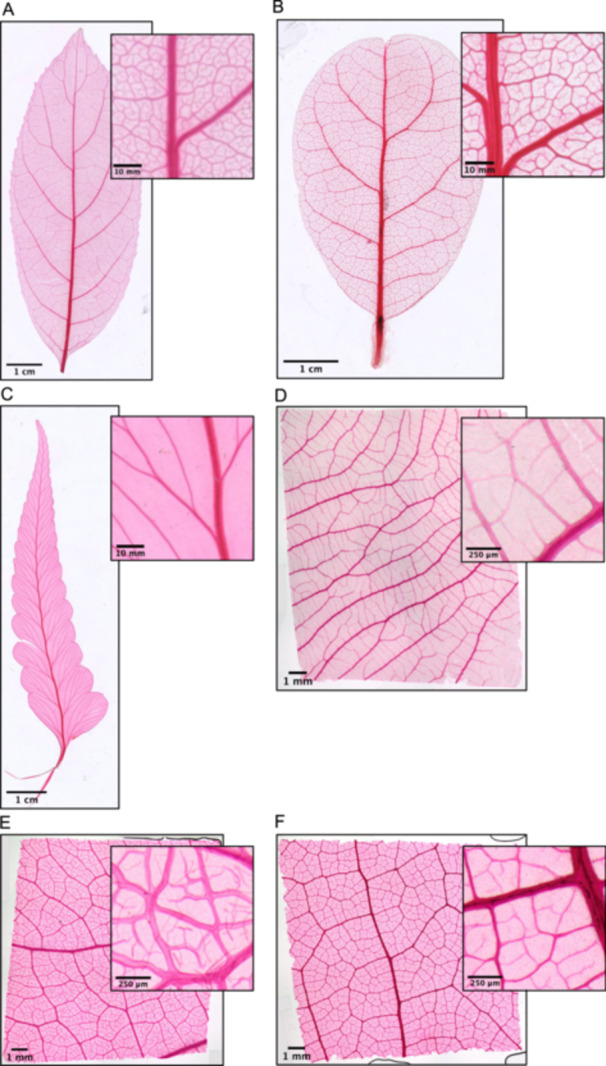
Examples of successfully imaged mounts. Inset photos are sized to a square according to their respective scale bar (e.g., 0.5 × 0.5 mm, 1 × 1 cm). (A) *Ehretia acuminata* R. Br. (Boraginaceae). (B) *Beilschmiedia tarairi* (A. Cunn.) Kirk (Lauraceae). (C) *Microlepia platyphylla* (D. Don) J. Sm. (Dennstaedtiaceae). (D) *Chionanthus pluriflorus* (Oleaceae). (E) *Diospyros chevalieri* De Wild. (Ebenaceae). (F) *Sloanea javanica* (Miq.) Szyszył. ex K. Schum. (Elaeocarpaceae). A–C were imaged via scanner; D–F were imaged via microscope.

A successfully processed and mounted sample will have an even gradient of dye intensity from midvein to minor veins with no tears or folds. Several example high‐quality images of mounts are shown in Figure 7.

#### Safety

The main risk of this protocol is chemical exposure, either via inhalation/contact with volatile solvents (ethanol, xylene) or via contact with bases (sodium hydroxide, sodium hypochlorite). Appropriate PPE is necessary, as is conducting steps involving volatile chemicals in a fume hood. There is also a risk of fire if hotplates are used and inappropriately placed near flammable chemicals, as well as the risk of environmental harm if waste materials are not disposed of appropriately. Details are provided in Appendix S3.

It is possible to replace the xylene‐based mounting medium with alternatives. Canada balsam has been proposed (Buechler, 2004), but also requires xylene, yellows over time, and shows autofluorescence (Ravikumar et al., 2014); it is therefore not recommended. Xylene can be replaced with toluene in the protocol to enable the use of toluene‐based mounting media (e.g., Permount; Thermo Fisher Scientific, Waltham, Massachusetts, USA); however, toluene has greater health risks (Blanc, 2018) and should not be used unless the desired mounting media are not available.

Aqueous/hydrocarbon/alcohol‐based mounting media like Euparal (Agar Scientific, Rotherham, United Kingdom), Histomount (Thermo Fisher Scientific), or Histo‐Clear II (National Diagnostics, Atlanta, Georgia, USA) have fewer safety concerns, reduce sample brittleness, and may be a good but more expensive alternative. They work well for slide mounts, but we are unsure of their performance for larger and thicker whole‐leaf mounts. In all cases, the protocol would remain the same through the staining step, after which alternative mounting methods and solvents would be used.

## CONCLUSIONS

### Limitations

The contrast provided by the staining comes from a lignin‐binding dye, safranin. Safranin was selected here because of its low cost, as well as for its useful fluorescence properties that enable single‐dye differentiation of lignin‐ and cellulose‐rich cell walls without counterstaining, as would be needed in brightfield imaging (Bond et al., 2008). However, other dyes can be used in place of or in addition to safranin to provide differential contrast (e.g., toluidine blue, fuchsin, or acridine orange) (Perdih and Perdih, 2011).

Very flimsy leaves do not do well with this protocol. They may be better processed using other published clearing protocols, e.g., with acids or chloral hydrate (Candela et al., 1999; García‐Gutiérrez et al., 2020). Very tough, thick, or well‐defended leaves are also difficult to process, because extensive lignification or secondary chemistry prevents digestion, resulting in limited contrast between vein and non‐vein tissues.

It is important to note that this protocol is resource intensive. High PPE usage is necessary due to the need to frequently change gloves and to clean workspaces and mounts. High chemical usage is also necessary due to the need to change out baths to ensure high mount quality.

The long‐term viability of mounts remains unclear. Contrast can be lost if mounts are exposed to excess sunlight or heat, so storage in dark and cool conditions is recommended. However, even under such conditions, the mounting medium may degrade over time. Protocols similar to this one were likely used to produce large collections of cleared leaves that are now archived in museums, some dating back to the mid‐20th century (e.g., the “Axelrod” collection at the University of California Museum of Paleontology). While the exact protocols used remain unknown, long‐term degradation of samples has occurred via oxidation and yellowing of the mounting medium from the edges, moving inward towards the center, sometimes obscuring the leaf (Appendix S2, Figure S2.7.1). Conserving these specimens has been difficult (Diane Erwin, curator of the University of California Museum of Paleontology, personal communication, December 2024). It is possible but not guaranteed that modern mounting media have better archival properties.

### Relevance

This protocol has made several contributions to the area of leaf clearing and mounting. Specifically, it provides detailed and contemporary guidance on laboratory setup and materials, as well as specific and illustrated instructions for leaf handling and addressing difficult cases and problems. Moreover, it highlights some of the key safety and sustainability issues relevant to implementation. This protocol therefore complements and extends extant protocols in ways that will facilitate and accelerate the production of high‐quality mounts in support of a range of research goals.

## AUTHOR CONTRIBUTIONS

B.W.B. and N.S. developed the initial protocol. I.N., L.M.T.A., M.A., and A.E. further developed the protocol; I.N. developed chemical processing methods, hazardous waste disposal, safety measures, and consolidated wet lab technique input from student wet lab workers. A.E., J.M., and B.V. developed imaging methods. I.S.M., M.B., L.M.T.A., S.C., and A.F. collected leaf samples. I.N., S.C., A.E., and A.F. wrote the initial draft and led the writing process. All authors contributed to editing all manuscript sections and figures, and all authors approved the final version of the manuscript.

## Supporting information


**Appendix S1.** Materials needed for the leaf clearing, mounting, and imaging protocol.
**Appendix S2.** Detailed protocol for leaf preparation, chemical clearing, staining, mounting, and imaging of leaves.
**Appendix S3.** Safety considerations.

## Data Availability

No supporting data were used.
