# Supplementary material for: A comprehensive illustrated protocol for clearing, mounting, and imaging leaf venation networks
Source: Appl Plant Sci. 2025 Mar 7;13(2):e70002. doi: 10.1002/aps3.70002 (PMC12038745; doi:10.1002/aps3.70002)
Supplement: Supplementary file 1 — Appendix S1. Materials needed for the leaf clearing, mounting, and imaging protocol. Appendix S2. Detailed protocol for leaf preparation, chemical clearing, staining, mounting, and imaging of leaves. Appendix S3. Safety considerations. [file APS3-13-e70002-s001.pdf]

DOI 10.1002/aps3.70002

APPENDICES

Appendix S1. Materials needed for the leaf clearing, mounting, and imaging protocol.

- 1.1. Lab space
- 1.2. Materials
  - 1.2.1. Durable supplies
  - 1.2.2. Consumable supplies
  - 1.2.3. Consumable chemicals
  - 1.2.4. Personal protective equipment
  - 1.2.5. Imaging technology
- 1.3. Constructing items marked with an asterisk (*)
  - 1.3.1. Making cassettes for large leaves
  - 1.3.2. Making cutting board tags

Appendix S2. Detailed protocol for leaf preparation, chemical clearing, staining, mounting, and imaging of leaves.

- 2.1 STEP 1 - Preparation
  - 2.1.1. Bath preparation
  - 2.1.2. Leaf and cassette preparation
  - 2.1.3. Challenging leaves
    - 2.1.3.1. Non-flat leaves
    - 2.1.3.2. Compound leaves
    - 2.1.3.3. Partial leaves
    - 2.1.3.4. Epidermal structures
    - 2.1.3.5. Difficult cases
- 2.2. STEP 2 - Clearing
  - 2.2.1. Leaf digestion
  - 2.2.2. Assessing progress
  - 2.2.3. Qualitative indications of progress
  - 2.2.4. Timing and troubleshooting
- 2.3. STEP 3 - Washing, bleaching, staining, and destaining
  - 2.3.1. Washing
  - 2.3.2. Bleaching
  - 2.3.3. Staining
  - 2.3.4. Destaining

DOI 10.1002/aps3.70002

2.4. STEP 4 - Mounting
2.4.1. Transferring samples between final baths
2.4.2. Preparing mounting materials
2.4.3. Making the mount
2.4.4. Large leaves
2.5. STEP 5 - Curing and finalizing mounts
2.5.1. Curing mounts
2.5.2. Air bubble removal and fixing other errors
2.5.2.1. Reinjection
2.5.2.2. Remounting
2.6. STEP 6 - Imaging
2.6.1. Scanner
2.6.2. Microscope and digital camera
2.6.3. Image stitching
2.7. STEP 7 - Long-term storage
Figure 2.7.1.
Appendix S3. Safety considerations.
3.1. PPE, engineering, and administrative controls
3.2. Hazardous waste disposal
3.2.1. Bath replacement
Figure 3.2.1.1.
3.2.2. Waste compatibility and disposal

DOI 10.1002/aps3.70002

Appendix S1. Materials needed for the leaf clearing, mounting, and imaging protocol.

1.1. Lab space

- Fume hood space
    - Option 1: Large fume hood, able to accommodate all containers containing volatile baths (minimum of 6 containers). Additionally, space for sample mounting requires 2-3 times the size of the sample, and space for tools plus space for curing.
    - Option 2: Two smaller fume hoods can be used as long as an airtight secondary container is used to transport samples from one fume hood to the other.
    - Option 3: One smaller fume hood can be used if necessary with proper rotation of dishes and secure airtight lids to a chemical storage cabinet outside the hood. This poses more safety hazards than keeping the volatile dishes in the fume hood at all times.
- Bench space: counter space large enough to accommodate the amount of digestion, washing, bleach bath containers. Additional workspace for sample preparation, note taking, sample digestion tracking, sink usage, and additional equipment is also required.
- Fire-resistant cabinet for storing flammable chemicals.

1.2. Materials

1.2.1. Durable supplies

- 8 borosilicate glass baths with matching close-fitting lids (Pyrex 234P dishes with 234-

DOI 10.1002/aps3.70002

PC lids are recommended).

- 2 round-ended tweezers (one for non-fume hood and one for fume-hood work)
- 2 metal spatulas (one for non-fume hood and one for fume-hood work)
- 1 lab trolley
- 1 scale
- 1 scissor
- 1 small paint brush / toothbrush
- 1 tape dispenser
- 1 metal tongs (~13 cm)
- small histology cassettes
- medium cassettes (hole size 0.5 cm or smaller)*
- large cassettes (hole size 1 cm or smaller)*
- 2 cutting boards (flexible thin plastic for lifting samples)*
- 1 plant press
- 1 dissecting microscope or magnifying lens
- 2 hot plates
- 1 timer
- 1 hole punch
- 1 graduated cylinder (1000 mL)
- 4 beakers: 2 × 500 mL, 2 × 1000 mL

1.2.2. Consumable supplies

- large sponge cut into approximately ~4 cm^2 cubes (for use with tongs with xylene

DOI 10.1002/aps3.70002

cleaning)

- cutting boards, thin plastic (for labels) *
- fishing line
- Paper, A4/Letter size (for visualizing digestion progress)
- dish sponges (for xylene cleaning [in fume hood], goggle cleaning, and regular dish cleaning; separate sponges for each task)
- painter’s tape
- lens paper (for cleaning mounts)
- paper towels
- weigh boats
- plastic transfer pipettes (~7 mL)
- razor blades
- Pencils

For acetate mounts:

- acetate sheets (Grafix brand recommended, not 3M)
- aluminum tape

For glass mounts:

- glass slides
- glass coverslips

DOI 10.1002/aps3.70002

1.2.3. Consumable chemicals

- ethanol, 95-100%, not denatured
- xylene
- xylene-based mounting medium (e.g., Fisher Healthcare PROTOCOL, Poly Sciences Poly-Mount Xylene)
- sodium hydroxide, solid pellets
- water, deionized
- water, tap
- sodium hypochlorite (household bleach), 5-10% in water
- safranin, powder

All of the above chemicals can be purchased at low purity levels and do not need to be analytic-grade, e.g., sodium hydroxide intended for soap-making, or bleach for household use.

1.2.4. Personal protective equipment

- nitrile gloves (thickest available, single-use)
- fire-resistant lab coats with cuffs
- lab goggles
- xylene-resistant gloves (fluoroelastomer, polyvinyl alcohol)
- face shields
- hair ties for securing long hair

DOI 10.1002/aps3.70002

1.2.5. Imaging technology

- Option 1 (for larger leaves, or where visualizing minor venation or finer anatomical features is not critical):
  - Flatbed scanner with trans-illumination capability and at least 1200 dpi resolution (e.g., Epson Perfection V850)
- Option 2 (for smaller leaves, or where visualizing minor venation or finer anatomical features is not critical):
  - Digital camera with 1× or 2× macro lens, or digital camera with compound/dissecting microscope
  - Trans-illumination (e.g., LED light table / light box, or microscope brightfield light source)
  - Fabric or plastic bag/hood to block external light
- Option 3 (for very small leaves, or where visualizing minor venation and fine anatomical features is critical):
  - Digital camera with compound or dissecting microscope with 2× - 40× objective
  - Trans-illumination (e.g., microscope brightfield light source)

1.3. Constructing items marked with an asterisk (*)

1.3.1. Making cassettes for large leaves

1. Cassettes can be made by cutting plastic seed germination trays or office organizers. Edges can be removed; excess height should also be removed so that cassettes will fit in glass baths.

DOI 10.1002/aps3.70002

Nylon and polypropylene have good resistance to xylene, while high-density polyethylene does not. Metal should not be used as it will corrode in sodium hydroxide. In practice, many plastics can be used so long as some degradation is acceptable.

1.3.2. Making cutting board tags

1. Take the thin plastic cutting board and cut off a strip of it, so the strip is 5 cm wide, and as long as the board.
2. Punch a series of holes down the long side of the strip, with each hole being made approximately every 1 cm.
3. Use scissors to cut between each hole, all the way through the strip, yielding 1 × 5 cm tags.
4. Cut a 12-15 cm piece of fishing line, and loop it through the hole.
5. Tie the loose ends together, so the tag is attached with a secured loop.
6. Put the fishing line loop (with tag attached) through a hole in the cassette, and thread the tag inside that loop emerging from the other side of the cassette hole. Later, the tag can be removed from the cassette (and put on a new one) without cutting the fishing line.

DOI 10.1002/aps3.70002

Appendix S2. Detailed protocol for leaf preparation, chemical clearing, staining, mounting, and imaging of leaves.

2.1 STEP 1 - Preparation

2.1.1. Bath preparation

Before preparing leaf samples, the following baths should be made in a borosilicate glass dish or chosen container with enough room to minimize overlap or contact between samples. When not in use, keep all container lids closed to prevent evaporation.

1. Digesting bath (sodium hydroxide 5% in water). Tap water can be used if it does not have a high mineral content. Sodium hydroxide for non-laboratory purposes can be used.
2. Washing bath (water). Tap water can be used; deionized is preferable. Use fresh water every day and discard after use.
3. Bleaching bath (sodium hypochlorite 2.5% in water). Household bleach can be used.
4. Dehydrating bath (ethanol 50% in water). Deionized water should be used.
5. Staining bath (safranin 0.1% in ethanol). Reagent-grade or food-grade ethanol can be used; denatured alcohol (i.e., methanol-diluted) cannot be used. 200-proof ethanol is preferable to 195-proof food-grade spirits (i.e., water-diluted).
6. Destaining bath (ethanol 100%). Ethanol purity as above.
7. Pre-mounting dilution bath (ethanol 50%, xylene 50%). Ethanol quality as above; reagent-grade xylene can be used.

DOI 10.1002/aps3.70002

8. Pre-mounting bath (xylene 100%). Xylene purity as above.

Baths 4-8 are volatile and should be prepared and handled in a fume hood. Xylene is hazardous and must be handled appropriately.

2.1.2. Leaf and cassette preparation

Choose a leaf with little to no damage, including herbivory, holes, mold, fungus, or tears. However, lower-quality samples can be used for test-runs to tune the protocol. While samples can be processed in batches, no two leaves or species will react the same way and it is important to expect variation and note differences in appropriate timings.

Place each leaf in a tissue cassette that is slightly larger in dimensions than the leaf. This provides gentle mechanical support for the whole leaf to rest upon, while allowing liquids to pass through the cassette (**Figure 2C**).

Histology cassettes can be directly labeled with a pencil. Tray cassettes can be labeled with a small piece of plastic (e.g., a piece of a thin cutting board) placed inside and/or attached with a fishing line. Labels should be made in pencil, as pen or marker may dissolve during the processing. If multiple samples share a single cassette, dissimilar samples should be placed within each cassette to avoid confusion. Brief label notes based on shape and size, but not color (as color will change throughout the process), can be added to each sample's tag to help visually distinguish samples.

DOI 10.1002/aps3.70002

2.1.3. Challenging leaves

2.1.3.1. Non-flat leaves

Leaves with three-dimensional structure (i.e., folding, wavy margins, thick midrib) pose a challenge during clearing and mounting stages. During digestion, non-flat leaves may be unevenly submerged, yielding uneven results or desiccation damage from air exposure. Folds and wavy margins can also cause tearing in the pre-mounting bath as it is difficult to unfold the leaf once it is rigid after destaining. During mounting, a non-flat leaf may become folded when flattened for mounting. Additionally, variation in thickness can cause air bubbles to form during mounting. To prevent these outcomes, press freshly collected leaves prior to digestion, then unfold them once softened in digestion baths. Samples that are folded prior to processing (e.g., herbarium samples, samples stored in small envelopes, etc.) can sometimes be unfolded successfully.

2.1.3.2. Compound leaves

Small compound leaves can be difficult to process because the rachis may become detached from the leaflets during digestion. Using mesh bags instead of histology cassettes may prevent leaf parts from floating away.

2.1.3.3. Partial leaves

If a leaf is highly damaged, or if an image of the whole leaf is not necessary, a small leaf section (e.g., 1 cm^2) can be excised using a razor blade, preferably on fresh tissue, taking care not to crack or tear the leaf while cutting by applying light pressure to the lamina adjacent to the cutting location. Fresh leaves cut more cleanly than dried leaves. Alternatively, damaged leaves can be

DOI 10.1002/aps3.70002

processed whole and only the subsection of interest can be imaged. Partial samples are useful for monocots with long leaves with parallel venation that does not change distally. Very large leaves may require impractically large containers, so these samples may be cut into pieces prior to analysis.

2.1.3.4. Epidermal structures

Trichomes and other epidermal structures obscure venation after clearing and should be removed by lightly pressing a rolled piece of painter's tape to the lamina, then quickly pulling it away. For leaves with dense trichomes, or thin delicate leaves, trichomes can be more carefully removed using brushes, blunt dissection tools, or tweezers, with the leaf placed under a dissection microscope for better visualization. This process requires a gentle hand to avoid leaf tearing. If trichomes cannot be removed during leaf preparation steps, digestion of the sample can be started and trichomes can be removed later as they soften.

Cuticles can also hinder digestion. To remove the cuticle, use a toothbrush with soft bristles or a sponge. Cuticles can also be removed via submersion in a chloroform bath located in the fume hood. Removal of the epidermis is also sometimes needed and can be carried out at the digestion stage; see Step 2.2.1, 'Leaf Digestion'.

Spore-bearing structures of ferns and lycophytes can also be removed prior to clearing as they obscure venation. Younger leaves are typically easier to work with in this case. The brush method described above is preferable to the tape method.

DOI 10.1002/aps3.70002

2.1.3.5. Difficult cases

Some species may not be successful using this protocol. Examples include those that are very flimsy (disintegrate in digestion or do not have sufficient lignification for good image contrast) or very sclerophyllous (too much lignification for good contrast). Also, species with additional structures (resin glands, punctations, crystals, or other vacuoles containing secondary compounds) can yield undesired contrast after clearing. Other protocols may be better used in these cases.

2.2. STEP 2 - Clearing

2.2.1. Leaf digestion

Gently place each cassette with the labeled sample in the digestion bath (**Figure 2A**). Fully submerge the sample in the bath by lightly shaking the cassette and/or flipping the leaf over and gently pushing it down with a piece of plastic cutting board. Ensure that no air bubbles are trapped underneath the leaf sample. Samples exposed to air eventually can fracture. If a sample was folded in storage, it can be unfolded gently with gloved hands after several hours of rehydration in the digestion bath. Unfolding must be done before staining steps to avoid uneven staining.

The digestion bath should be left covered. It can be left at room temperature, or the digestion process can be accelerated by placing the bath on a hotplate set to a temperature between 40-50°C or by leaving the bath in a warm room. High temperatures also can digest samples too

DOI 10.1002/aps3.70002

quickly. Starting digestion before the weekend is not recommended, as fragile samples can disintegrate in 2-3 days.

Digestion can also be accelerated by using a rocker/shaker table or hand-agitating the bath regularly, gently tapping the loose epidermis with a gloved fingertip, softly brushing the sample's surface with a dampened fine-tipped paint brush. Be aware that these measures can also damage the leaf. For some samples, it is possible to completely remove the leaf epidermis during the digestion step by gently tapping across the leaf, which enables better visualization of minor veins. If a sample has a layer of epidermis that peels naturally during digestion, then tweezers can be used to grasp the epidermis and to gently peeled it off. In sclerophyllous leaves, brushing the leaf surface with a paintbrush or toothbrush can also help to slough off the epidermis, especially if it comes off in smaller chunks rather than in a single layer. If the leaf begins to tear, stop trying to separate the epidermis and let the sample digest longer before attempting further epidermis removal. In some species it is difficult or impossible to remove the epidermis. Blister-like formations may occur on leaves during the digestion process and cause uneven digestion. Those blisters are often filled with a darker-colored digestion bath, trapped under the epidermis. The blisters can be gently released with applied pressure to the leaf using a gloved finger.

2.2.2. Assessing progress

Samples should be checked daily for digestion progress. Delicate samples may require more frequent observation. Typically, all but the flimsiest samples can be left to digest over a weekend at room temperature.

DOI 10.1002/aps3.70002

When checking samples, gently lift the cassette with the leaf out of the digestion bath to observe the sample's color, opacity, and thickness of the epidermis (**Figure 3F**). A strong light source is useful for assessing sample state. Sliding white paper under the dish can also help to give contrast for visualizing changes. Delicate samples can be manipulated by using a flexible plastic cutting board to lift from underneath. Stronger samples may withstand being manipulated with tweezers or gloved hands. Limit the amount of time the samples are out of the bath to avoid desiccation. A quick glance should be sufficient for most samples; longer observations can be made after transferring samples to a water bath.

2.2.3. Qualitative indications of progress

Samples initially will have a dark brown or black color. This darkening will shift over time to lightening, allowing the digestion progress to become visible. Dark colors should fully disappear before digestion is complete (**Figure 3A-E**). However, some samples, especially larger and/or thicker ones, may still have dark coloration inside of their major veins once digestion of the lamina is completed; this coloration is not typically problematic during imaging. Waiting too long for major veins to clear can cause disintegration of the lamina.

Almost-digested samples are lighter in color and more translucent. The outer epidermal tissue may begin to loosen and shed, contributing to changes in translucency and thickness. Samples may appear patchy as pieces of outer epidermis shed unevenly.

DOI 10.1002/aps3.70002

Fully digested samples are evenly translucent, lightly colored, and clear of epidermal tissue. Cloudy or splotchy samples are usually not yet ready (**Figure 3G**). However, an uneven appearance may occur in some species or in leaves with damage from herbivory or fungi.

Over-digested samples are tattered, torn, and sensitive to handling (**Figure 3H**). They can be difficult to maneuver without causing physical damage. In extreme cases, the leaf may completely disintegrate. To minimize losses, inspect progress regularly. Avoiding over-digestion is more important than all other criteria.

2.2.4. Timing and troubleshooting

The time required for digestion varies by species, and is typically much longer for thick or sclerophyllized leaves. Less lignified leaves may require 1-2 days at room temperature while more lignified leaves may require 3-4 weeks with mild heating. Leaves of the same species may respond to digestion differently. Processing multiple samples of the same species and experimenting with digestion timing can be useful, as can keeping a notebook or photographic records of progress.

2.3. STEP 3 - Washing, bleaching, staining, and destaining

2.3.1. Washing

Transfer digested samples to the washing bath by quickly moving them in cassettes, gently shaking out any residual liquid prior to transfer. Leave in the washing bath for 1-2 minutes. The

DOI 10.1002/aps3.70002

bath can be gently rocked to increase flow over the sample. Samples cannot be stored in the washing bath, even overnight, because they are extremely fragile at this stage.

If multiple samples are in a single cassette, and some are not ready for transfer, separate them in the washing bath by lifting from below with a piece of plastic cutting board, and transfer into a separate cassette.

2.3.2. Bleaching

Samples that are already white or transparent after digestion do not require the bleaching step. All other washed samples should be transferred to the bleaching bath. Samples should stay in the bleaching bath for up to 20 minutes (typically 1-2 minutes), until they lose their color and become pale yellow or white (**Figure 3I-J**). Very delicate leaves may become nearly transparent. Check the samples every few minutes, as leaving samples for too long can destroy them. Samples still continue to slowly bleach even when placed back into the water bath; this process can be useful for delicate leaves. If the sample does not fully turn white or transparent, or if it bleaches in uneven patches, move it back to the washing bath, and then to the digestion bath for further digestion.

For very sclerophyllous samples, mild bleaching of the sample earlier in digestion allows for better visualization. Samples can be bleached and then assessed for translucency, pigmentation, and amount of epidermal tissue, then returned to the digestion bath if needed. However, this process should generally be avoided as it can cause excess damage to the sample.

DOI 10.1002/aps3.70002

After bleaching, move the sample from the bleaching bath back to the washing bath for 1-2 minutes and apply gentle agitation.

2.3.3. Staining

These steps are carried out in a fume hood for safety (**Figure 2B**). All baths should have closed lids except during sample transfers to prevent solvent evaporation. Samples should be transferred quickly to avoid tissue damage due to desiccation. Place the sample in the dehydrating bath and gently rock the bath for at least 1 minute. Then transfer the sample from the dehydrating bath to the staining bath and leave it for 40-50 minutes. Some samples (e.g., thicker leaves) may take longer to stain, so staining time may need to be adjusted. A well-stained sample should appear very dark pink or deep red in color.

2.3.4. Destaining

Next, transfer the sample to the destaining bath for at least 1 hour. Longer times might be required for samples that took longer to stain. Samples should be de-stained to a pink color with the veins taking a dark red color, and no patchy coloration. The stain contrast and intensity will vary across samples according to leaf thickness and vein density. Samples that are not satisfactorily stained can be re-stained by moving them back to the safranin bath until sufficiently stained. Patchy coloration reflects incomplete digestion and requires moving the sample back through dehydration and washing to digestion, or starting over with new samples.

DOI 10.1002/aps3.70002

Samples can be left in the destaining bath for up to 1-2 days; longer times can yield complete destaining. If the destaining bath becomes contaminated by a large concentration of dye, it can be used to store samples for longer periods of time.

2.4. STEP 4 - Mounting

2.4.1. Transferring samples between final baths

These steps are also carried out in a fume hood for safety.

Transfer cassettes to the pre-mounting dilution bath for 1-2 minutes with gentle rocking. Then transfer samples to the pre-mounting bath. Leave samples in this bath for at least 1 minute, with gentle rocking. The sample may become rigid during this or the previous step; attempting to uncurl a leaf at this stage can cause fracturing. Samples can be stored in this bath indefinitely. However, care must be taken to ensure the samples are always fully submerged and weighed down in a flat position. Evaporation of the bath over time will cause the samples to desiccate, curl, and fracture.

Transfers between baths containing ethanol and xylene should be rapid. Evaporation of solvent from samples at this stage can cause desiccation, damage, and poor image quality.

2.4.2. Preparing mounting materials

Mounting must be done in a fume hood for safety. Prepare two transparent rectangles, a plastic Pasteur pipette, mounting medium, and paper towels within the hood. The below protocol

DOI 10.1002/aps3.70002

assumes the rectangles are acetate sheets, but glass, acrylic (plexiglass), or polycarbonate (each typically of 1-3 mm thickness) can also be used to create archival-quality mounts. For small samples, glass slides and coverslips can be used instead. Mounting medium should be xylene-based, e.g., ‘Healthcare PROTOCOL’.

Keep a clean beaker to hold tools (xylene tongs, chemical-resistant marker, tweezers, scissors, spatula, bent dissection probe) nearby. The hood must be dust-free; dust caught in mounts cannot be removed and interferes with image quality. Always clean the mounting area with pure 100% xylene after completing each mount (or even before completing a mount by wiping it with xylene-soaked lens paper) so that the surface of the newly finished mount doesn't get dirty and sticky. Also pre-dispense the tape later needed for edge sealing. This minimizes stress on the gloves, which helps minimize them tearing and ultimately helps prevent chemical exposures.

Acetate sheets should be sized such that the leaf being mounted is provided a wide margin along each side; for smaller samples (i.e., samples ≤5 cm), the acetate should be at least 2-3 cm longer on each side than the leaf to be mounted. For larger samples, be especially generous when deciding the size for acetate sheets; the extra width is critical for preventing air bubbles from entering. Extra width is also necessary for leaves with large, protruding, or angular primary veins that cause sheets to pull away from each other.

2.4.3. Making the mount

After preparing all the mounting materials, use a transfer pipette to add the mounting medium to the middle of the acetate sheet, i.e., the area where the center of the leaf will lay (**Figure 4A**).

DOI 10.1002/aps3.70002

The amount of medium used will depend on the size of the leaf. A 7 mL pipette is sufficient for the first side of a 10-15 cm leaf. Pipette slowly to avoid creating bubbles. Pick up the sample by the petiole or near the base (if the leaf is missing its petiole) using tweezers, tongs, and/or a flexible plastic cutting board segment, then place on the mounting medium. When working with large leaves or leaves with a thick midrib, the petiole can be removed with scissors. By shortening or completely removing the petiole, the mount is less likely to form air bubbles. Do not remove the petiole in earlier steps, as it can be a useful handle for lifting samples.

Next use the pipette to place more mounting medium on top of the sample. Make sure to cover the whole sample. Uncovered medium will quickly cure, so do not leave the sample exposed to air. Align another equally sized acetate sheet on one edge of the first sheet and slowly lower the sheet over the sample while steadily pushing out air bubbles (**Figure 4B**).

If bubbles appear, partially peel the sheets apart, use the pipette to inject additional mounting medium, and repeat the process. Be careful to avoid excess mounting medium squeezing out the sides of the mounts where it can contaminate gloved hands and/or smear on the fronts of the sheets. If such contamination does occur, change gloves and use lens paper to sponge away excess mounting medium. Soaking paper in xylene and/or ethanol first may help.

Once the leaf is mounted between the two sheets, remaining air bubbles can be removed by gently pushing them to the edges of the acetate sheet with the palm of the gloved hand. The mounting medium should now be spread across the whole sheet with residual air bubbles pushed

DOI 10.1002/aps3.70002

to the side or removed completely if possible. Any spillover of excess medium from the mount should be wiped away immediately with paper towels.

After removing large air bubbles and spreading the mounting medium, the mount should be sealed with metal tape along all four edges. This prevents air intrusion during curing and reduces long-term degradation of the cured mounting medium.

For glass slides, the mounting process is similar. Grasp coverslips and slides only by the edges, to avoid smudging. Place 1-2 drops on a slide using a pipette. Using tweezers and/or a spatula, gently pick up the sample, being careful to not damage it by pinching. Place it directly on the mounting medium. Then place one edge of a glass coverslip to the side of the sample. Slowly lay it down over the sample with even pressure, while pushing any air bubbles out. Air bubbles, if formed, cannot easily be removed from glass. Sealing is not feasible for glass slides.

Clean any mounting medium that has smudged the acetate or glass with lens paper (more abrasive materials such as a paper towel will scratch acetate or plexiglass) and xylene. Xylene and/or ethanol can also be used to clean surfaces (e.g., bottom of fume hood, tools, etc.) that have mounting medium spillage. Using tongs to manipulate sponges or lens paper will reduce xylene exposure on gloves. Excess medium can be wiped off of xylene-resistant gloves using pure xylene. However, if the mounting medium gets on the nitrile gloves, change them out immediately.

DOI 10.1002/aps3.70002

Use a pencil or laser-printed labels to label the mounted samples. Do not use a pen, markers, or an inkjet printer for labeling, because if samples require any later cleaning, solvents can dissolve the ink.

2.4.4. Large leaves

Large leaves require extra care in mounting on acetate due to the difficulty of handling materials.

The sample must be rigid enough to move and then mount. If the sample has any fold lines, pay extra close attention to their condition as they can tear further during the mounting process. Handling large samples without tearing may require two or more people to work together.

Prepare a whole bottle of mounting medium, tweezers, wooden stir sticks, beakers, acetate sheet, xylene tweezer tool, paper towels, tape, and scissors. Place the acetate on a clean paper towel then carefully pour out some mounting medium onto the acetate. The amount of medium on the acetate sheet should be enough such that when the leaf is placed on top, the entire bottom surface is in contact with the medium. Maneuver the sample into the desired place and/or gently remove any folding/other complications that happened during initial placement using stir sticks. Then pour out more mounting medium to cover the top half of the leaf. Make sure to use enough to fill any voids in the mount caused by a thick leaf midrib. Then, using a pipette, place extra mounting medium onto the parts of the acetate that do not have any. The amount should be just enough to minimize the risk of too little medium causing air bubbles.

DOI 10.1002/aps3.70002

Take a second piece of acetate and, starting at one end or side, carefully start lowering the acetate sheet onto the sample and first sheet. Start cleaning up the excess mounting medium that leaks from the sides of the acetate with the tongs and a paper towel or sponge soaked in xylene. It may be necessary to add more mounting medium at this stage by slightly opening the two sheets and using a pipette to pour more in. Try to avoid this by using enough mounting medium at the start. Start taping the mount closed. This may have to be done in smaller sections (~15 cm at a time) if the whole length at once is too unwieldy. Once the mount is fully sealed, wash the outside with xylene-soaked lens wipes using tongs, wipe again with dry lens paper, and set aside to dry. Clean the fume hood of excess mounting medium that may have leaked out.

Due to the amount of medium used, the mount should remain in the fume hood for at least a week before checking for completion of curing. Place the mount between cardboard sheets under weights or in a plant press to ensure it cures flat.

2.5. STEP 5 - Curing and finalizing mounts

2.5.1. Curing mounts

Samples should remain flat in the fume hood during curing.

Samples are partially cured when the mounting medium has solidified around the edges of the mount. Fully cured mounts have all of the mounting medium solidified. Samples can be gently heated to 30-40°C to speed the curing process in the fume hood by placing them on a hotplate

DOI 10.1002/aps3.70002

with a piece of fabric or cardboard to support them. Extreme care should be taken if heating mounts, because xylene is flammable.

After the mounts have fully cured, the sample should appear fully transparent with veins appearing in pink (**Figure 4C-D**). Sample transparency and contrast increases continuously during the curing process. Final curing may take several weeks depending on the amount of mounting medium used. After removal from the fume hood, there should be no odor of volatile chemicals; any odoriferous samples should be immediately returned to the hood for safety.

2.5.2. Air bubble removal and fixing other errors

For incompletely cured mounts, air bubbles can often be moved to the side of the leaf, so as not to interfere with sample imaging. To fully remove those air bubbles, first place a large sheet of lens paper down in the fume hood. Then, place the mounted sample on top of the lens paper so that the finished mount does not get scratched. To remove small air bubbles that are in the way of the leaf, begin by slowly pushing them towards the edges of the acetate sheet with a gloved palm. Push in a gentle yet firm rolling motion. Excess pressure can inadvertently damage the sample.

Over time, air bubbles can migrate into the leaf and disrupt image quality (**Figure 4E**). This is caused by inadequate amounts of mounting medium, or by using an acetate sheet that is too small relative to the leaf size. Leaves with these types of air pockets should be returned to the fume hood for reinjection of mounting medium or remounting on larger sheets. Remounting should be done as soon as possible to prevent permanent damage to the sample.

DOI 10.1002/aps3.70002

2.5.2.1. Reinjection

In the fume hood, cut off one corner of the mount, ideally the one closest to the petiole. Then, insert a fresh pipette full of mounting medium inside the cut end and directly inject more mounting medium into the original mount. Push away air pockets and reseal the mount. It can help to hold the mount vertically with the cut end up while doing this, so that gravity pulls the mounting medium down and buoyancy pulls air bubbles out. Re-seal edges with tape and proceed with curing.

2.5.2.2. Remounting

If the petiole warps the acetate sheets, air bubbles cannot be easily resolved by reinjection. For these cases, larger mounts should be used. Cut around all sealed edges of the old mount and transfer the leaf and mount into the pre-mounting bath, letting it reabsorb the xylene overnight. Then peel the mount away and repeat the mounting process. The pre-mounting bath will need changing after this action.

For glass slides, the entire mount should be submerged in xylene for 2-3 days until the glass slide cover loosens; then the mounting process can be repeated.

Remounting and/or reinjection may lead to ripping or destroying of a reasonable-quality sample. If air bubbles have been present for a long time, sample desiccation may have occurred, also limiting the quality improvement possible via remounting.

DOI 10.1002/aps3.70002

2.6. STEP 6 - Imaging

Imaging should be carried out after complete curing. Imaging uncured samples risks chemical spills. However, mounts may whiten or crack after very long storage (multiple years to decades, less if kept in warm or sunlit conditions) so imaging should not be delayed unnecessarily.

2.6.1. Scanner

Imaging with a scanner that uses trans-illumination (e.g., for film, Epson V850) is the easiest and most suitable method for mounts of all sizes (**Figure 6A**). A scanner that uses reflective illumination (e.g., for documents) is not suitable. The mount should be placed on the scanner bed with the light source configured for trans-illumination (‘positive film’ mode on many scanners). Mounts that do not lay flat may need a heavy weight pressed on top of the scanner to ensure that the leaf lays on a consistent focal plane. The scanner should be set to 16 bits/channel (48-bit color) mode. In most species, 1200 dpi is sufficient to visualize most veins, but 6400 dpi is needed to ensure minor veins are visible. Very large leaves that do not fit onto the scanner bed can be scanned in segments by manually moving the sample between scans.

2.6.2. Microscope and digital camera

A 1:1 macro lens coupled to a digital camera can achieve resolution slightly better than a scanner, and a compound or dissecting microscope can achieve substantially higher resolution. Imaging large extents is difficult with a microscope due to the higher magnification, so this approach is most suitable for slide-mounted leaves unless a computerized translation stage is also

DOI 10.1002/aps3.70002

available. If a macro lens and camera is used, it should be located above the mount on a copy stand.

In both cases, mounts should be trans-illuminated. To trans-illuminate images taken with a macro lens or dissecting microscope, a light table can be placed beneath the mount (**Figure 6B-C**). For a compound microscope, use the brightfield light source. Minimize other light sources which can cause reflection artifacts. Camera or microscope aperture should be set to approximately f/8 as a tradeoff between sharpness and depth-of-field. A low ISO should be used, which may require a slower shutter speed. Vibrations should be minimized by placing the whole apparatus on a stable surface or on a rubberized mat. If possible, trigger the camera remotely and on time-delay to avoid vibrations from hand contact.

2.6.3. Image stitching

Samples that cannot be imaged in a single camera frame require image stitching to yield a complete mosaic. Ensure images are obtained under identical lighting/resolution settings and have at least 30% overlap in each direction to facilitate stitching. Images can then be stitched together using free panorama software, e.g., Pandora (https://shallowsky.com/software/pandora/, plugin available for GIMP) or MosaicJ (https://bigwww.epfl.ch/thevenaz/mosaicj/, plugin available for ImageJ/FIJI), or Hugin (https://hugin.sourceforge.io/).

For samples that were physically separated into pieces prior to imaging (due to large size or damage during processing) the above approaches will not work well due to lack of image

DOI 10.1002/aps3.70002

overlap; instead, manual alignment in an image editor is necessary, or the result must be accepted as-is.

2.7. STEP 7 - Long-term storage

After samples are cured, they can be stored at cool or room temperatures in dark conditions. Extended light or heat exposure can degrade stain contrast.

Incompletely cured mounts may appear cured while still having liquid mounting medium present, so proper personal protective equipment should be used when initially handling stored mounts.

Acetate sheet mounts can be stored between cardboard spacers in a plant press. Glass or plastic mounts can be stored in a slide box.

Over multiple years or decades, oxidation or other chemical degradation processes affecting the cured mounting medium can lead to whitening or powderization of the mounting medium and/or leaf (**Appendix 2**, **Figure 2.7.1**). Use of glass or plexiglass may yield longer archival lifetimes than acetate.

DOI 10.1002/aps3.70002

Figure 2.7.1.

An example degraded mount from the University of California Museum of Paleontology collection (‘ID744’). This sample is mounted between plexiglass plates and was produced using an unknown protocol several decades ago. Note the slow oxidative damage around the sample.

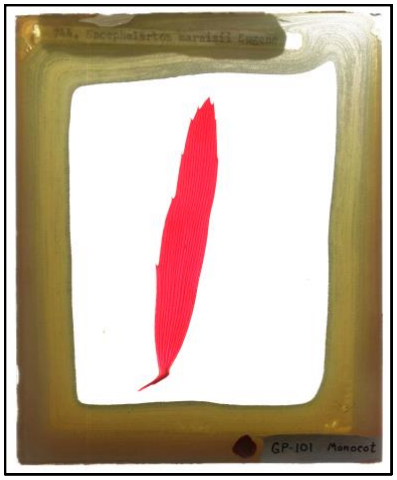

DOI 10.1002/aps3.70002

Appendix S3. Safety considerations.

3.1. PPE, engineering, and administrative controls

Hotplates pose a fire risk. If the hotplate is set too hot and left unattended, the digestion bath will evaporate fully. This can result in the loss of the samples and a potential fire. Additionally, if additional cold water is re-added to a hot glass container, the container may shatter and create glass shard projectiles.

Sodium hydroxide and sodium hypochlorite (bleach) are both corrosive and can cause skin irritation or chemical burns upon contact (Riddick, 2020). Appropriate safety controls include personal protective equipment (PPE), such as nitrile gloves, lab coats, face shields, and safety goggles. Cuffed lab coats should be used, as open-sleeved coats allow for inadvertent dipping into the open baths of chemicals, as well as arm exposure to stray droplets.

Ethanol, xylene, and the xylene-based mounting medium are all volatile and flammable. They can cause skin irritation upon contact, and can cause headaches, dizziness, muscle weakness, and confusion upon inhalation (Langman, 1994). Xylene residues will smear and spread; these residues can be cleaned using ethanol. Appropriate safety controls include PPE (nitrile gloves, lab coats, goggles, face shields) and engineering controls (handling only within a fume hood, eliminating all ignition and heat sources from the work location).

DOI 10.1002/aps3.70002

Engineering controls (i.e., changing the physical organization of the workspace to enhance safety by isolating hazards) should also be considered to mitigate risks inside the fume hood. Limiting the number of baths being used can improve maneuverability within the fume hood, but may reduce sample processing throughout, as well as the maximum leaf size that can be processed. Arranging supplies within the fume hood before starting work can also minimize the risk of spills, cuts, and smears. Heated digestion baths should be kept out of the fume hood to avoid potential ignition of volatile chemicals.

Administrative controls (i.e., developing lab procedures and policies to reduce hazards and likelihood of harmful exposure) should also be considered when training on the protocol. The highest-risk components of the protocol (e.g., preparing and disposing of volatile baths, moving baths, mounting leaves), and potential symptoms of chemical exposure should be clearly communicated.

There are limits to the value of PPE in this protocol. Xylene breakthrough time on nitrile gloves is on the order of minutes (Packham, 2006). Double gloves for all hazardous fume hood work are recommended, and gloves should be changed promptly after contact with xylene. Bulkier xylene-resistant gloves (fluoroelastomer, polyvinyl alcohol) for mounting can also be used, but will reduce dexterity.

REFERENCES

Langman, J. M. 1994. Xylene: Its toxicity, measurement of exposure levels, absorption, metabolism and clearance. *Pathology* 26: 301–309.

DOI 10.1002/aps3.70002

Packham, C. 2006. Gloves as chemical protection - can they really work? *The Annals of Occupational Hygiene* 50: 545–548.

Riddick, J. M. 2020. Hazards of sodium hydroxide. *Loss Prevention Bulletin* 271.

3.2. Hazardous waste disposal

3.2.1. Bath replacement

The washing bath should be changed daily. The digestion bath loses potency over time. It should be replaced when its color becomes brown, cloudy, or when there is excess epidermal tissue floating in it (**Appendix 3, Figure 3.2.1**). This typically occurs after 1-2 weeks of constant usage. The bleaching bath should be changed if it loses its potency or when it has excess epidermal tissue. The dehydrating bath should be changed every few weeks, if a substantial fraction of the ethanol evaporates, or if there is tissue collecting in the bottom of the dish. The staining bath should be changed at a similar frequency, or if the image quality of processed samples begins to decrease (e.g., low contrast, blotchy contrast, or high particulate contamination). The destaining bath should be changed when it becomes opaque. However, a new destaining bath can overly destain samples quickly, so working with a mildly used (e.g., light pink) destaining bath will reduce this risk. Discarded liquid can either be re-used to make the staining bath or discarded.

DOI 10.1002/aps3.70002

Figure 3.2.1.1.

A digestion bath that needs changing (note leaf debris in bottom).

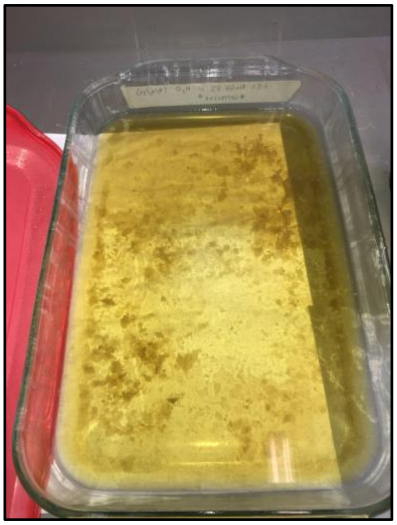

DOI 10.1002/aps3.70002

3.2.2. Waste compatibility and disposal

The used washing bath can be disposed of in drains. Samples that fail at the bleaching or digesting stage can be discarded as regular trash.

All other baths and samples that fail at the dehydrating or later stages should be treated as hazardous waste.

All volatile liquid wastes (dehydrating, staining, destaining, pre-mounting dilution, and mounting baths) can be stored safely together. Care should be taken due to the volatile and flammable nature of this waste.

All non-volatile liquid wastes (digesting, bleaching baths) can be stored and disposed of together. Care should be taken due to the corrosive nature of this waste.

All solid wastes (e.g., gloves, pipettes, paper towels) can be stored and disposed of together. Such waste should not be removed from the fume hood if volatile chemicals are still present on it.

Cassettes, baths, and other durable materials can be re-used repeatedly after cleaning and do not need to be discarded. Materials used in xylene can be cleaned with ethanol; all other tools can be cleaned with soap and water.
